# The ATRA-induced differentiation of medulloblastoma cells is enhanced with LOX/COX inhibitors: an analysis of gene expression

**DOI:** 10.1186/1475-2867-14-51

**Published:** 2014-06-13

**Authors:** Petr Chlapek, Jakub Neradil, Martina Redova, Karel Zitterbart, Jaroslav Sterba, Renata Veselska

**Affiliations:** 1 Department of Experimental Biology - Laboratory of Tumor Biology, School of Science, Masaryk University, Kotlarska 2, 611 37 Brno, Czech Republic; 2 Department of Pediatric Oncology, University Hospital Brno and School of Medicine, Masaryk University, Cernopolni 9, 613 00 Brno, Czech Republic; 3 Masaryk Memorial Cancer Institute, Zluty kopec 7, 656 53 Brno, Czech Republic

**Keywords:** All-*trans* retinoic acid, Caffeic acid, Celecoxib, Medullobastoma, LOX and COX inhibitors

## Abstract

**Background:**

A detailed analysis of the expression of 440 cancer-related genes was performed after the combined treatment of medulloblastoma cells with all-*trans* retinoic acid (ATRA) and inhibitors of lipoxygenases (LOX) and cyclooxygenases (COX). The combinations of retinoids and celecoxib as a COX-2 inhibitor were reported to be effective in some regimens of metronomic therapy of relapsed solid tumors with poor prognosis. Our previous findings on neuroblastoma cells using expression profiling showed that LOX/COX inhibitors have the capability of enhancing the differentiating action of ATRA. Presented study focused on the continuation of our previous work to confirm the possibility of enhancing ATRA-induced cell differentiation in these cell lines via the application of LOX/COX inhibitors. This study provides more detailed information concerning the mechanisms of the enhancement of the ATRA-induced differentiation of medulloblastoma cells.

**Methods:**

The Daoy and D283 Med medulloblastoma cell lines were chosen for this study. Caffeic acid (an inhibitor of 5-LOX) and celecoxib (an inhibitor on COX-2) were used in combined treatment with ATRA. The expression profiling was performed using Human Cancer Oligo GEArray membranes, and the most promising results were verified using RT-PCR.

**Results:**

The expression profiling of the selected cancer-related genes clearly confirmed that the differentiating effects of ATRA should be enhanced via its combined administration with caffeic acid or celecoxib. This effect was detected in both cell lines. An increased expression of the genes that encoded the proteins participating in induced differentiation and cytoskeleton remodeling was detected in both cell lines in a concentration-dependent manner. This effect was also observed for the *CDKN1A* gene encoding the p21 protein, which is an important regulator of the cell cycle, and for the genes encoding proteins that are associated with proteasome activity. Furthermore, our results showed that D283 Med cells are significantly more sensitive to treatment with ATRA alone than Daoy cells.

**Conclusions:**

The obtained results on medulloblastoma cell lines are in accordance with our previous findings on neuroblastoma cells and confirm our hypothesis concerning the common mechanism of the enhancement of ATRA-induced cell differentiation in various types of pediatric solid tumors.

## Background

Medulloblastoma (MBL), an embryonal neuroectodermal tumor of the cerebellum, is the most common type of malignant brain tumor in children. Although recent advances in MBL therapy have led to a dramatic increase in survival rate, the mortality rate is currently approximately 20–40% [1]. Moreover, MBL survivors are often affected by treatment-related side effects such as growth hormone deficiency, gonadal alterations, hypo- or hyperthyroidism, and long-term cognitive, neuropsychological and academic impairments, etc. New approaches are thus needed to improve the survival rate and to reduce the negative side effects of MBL treatment [2].

The induced differentiation of tumor cells has become a promising strategy in modern antineoplastic therapy. Retinoids, which are derivatives of vitamin A, are the most frequently used group of cell differentiation inducers. The regulation of relevant cell signaling pathways via retinoids is based on the activation of two groups of nuclear receptors, RAR and RXR [3,4]. These activated receptors can influence the transcription either directly by binding to the DNA or indirectly by interacting with other transcription factors [5,6].

In general, retinoids play an important role in cell proliferation and differentiation, and their efficacy in the treatment of various types of tumor cells has been described both *in vivo* and *in vitro*[7-11]. However, the toxicity of and intrinsic or acquired resistance to retinoids substantially limit their use in clinical protocols [12].

Therefore, special attention has been paid to treating cancer cells with a combination of retinoids and other compounds that may enhance or prolong their antineoplastic effects. The enhancing effects of these modulators were described in several clinical trials focused on the treatment of leukemia [13-16]; they were also demonstrated under *in vitro* conditions using tumor cells of a neurogenic origin [17-20].

To date, many studies on various cancer cell lines have reported the additive or synergistic effects of combined treatment with retinoids and inhibitors of lipoxygenases (LOX) [21-24] or cyclooxygenases (COX) [25-27]. The molecular mechanisms of this modulation remain unknown, but the published data suggest the inhibition of the retinoid degradation pathways [28] or the cooperation of compounds that are utilized in cell signaling inhibition (through the PI3K/Akt pathway) or the induction of the mitochondrial apoptotic pathway [25].

Our previous studies were also focused on how to enhance the differentiation effect of all-*trans* retinoic acid (ATRA) through its combination with LOX/COX inhibitors in neuroblastoma cell lines [20,29]. In these experiments, we used caffeic acid (CA) as an inhibitor of 5-LOX and celecoxib (CX) as an inhibitor of COX-2. Our results clearly confirmed that the ATRA-induced differentiation of neuroblastoma cells can be enhanced via the combined application of these inhibitors [20,29]. Furthermore, data from the expression profiling of the treated cells showed an increase in the expression of the genes involved in the process of retinoid-induced neuronal differentiation, especially in cytoskeleton remodeling after combined treatment [20].

To verify these findings, we used a different type of neurogenic tumor cells, i.e. established medulloblastoma cell lines but the same experimental design for the treatment of these cells. Our new data from the gene expression profiling of medulloblastoma cells also demonstrated the capability of CA or CX to enhance the cell differentiation induced via ATRA.

## Methods

### Cell lines and cell culture

Daoy (ATCC HTB-186™) and D283 Med (ATCC HTB-185™) medulloblastoma cell lines were used in this study. These cell lines were chosen according their different origin (primary tumor vs. metastatic site) and their different biological features as described by supplier in the documentation to cover known heterogeneity in this disease. Both of these cell lines are widely used in medulloblastoma research. These cell lines were maintained in Dulbecco’s modified Eagle’s medium (DMEM)/Ham’s F-12 medium mixture (1:1) supplemented with 20% fetal calf serum, 1% non-essential amino acids, 2 mM L-glutamine, 100 IU.ml^-1^ penicillin, and 100 mg.ml^-1^ streptomycin (all purchased from PAA Laboratories, Linz, Austria). Cell culture was performed under standard conditions at 37°C in a humidified atmosphere containing 5% CO_2_. Both of these cell lines were subcultured 1–2 times weekly. The Research Ethics Committee of the University Hospital Brno approved the study protocol.

### Chemicals

ATRA (Sigma-Aldrich, St. Louis, MO, USA), CA (Sigma), and CX (LKT Laboratories, St. Paul, MN, USA) were prepared as stock solutions at concentrations of 100 mM in dimethyl sulfoxide (DMSO) (Sigma). For experiments, these stock solutions were diluted in fresh cell culture medium to obtain final concentrations as follow: 0.05 and 0.1 μM of ATRA for the treatment of D283 Med cells, 1 and 10 μM of ATRA for the treatment of Daoy cells, 13 and 52 μM of CA, as well as 10 and 50 μM of CX for the treatment of both cell lines.

### Experimental design

The experimental design was the same as that in our previous studies [20,29]: the cell populations were treated with ATRA alone or with ATRA and an inhibitor (CA or CX) at the concentrations mentioned above. The concentrations of ATRA and inhibitors were chosen on the basis of previously published data, and they corresponded to the plasma levels obtained using these compounds therapeutically [23,30-33]. However, lower concentrations of 0.05 and 0.1 μM ATRA were used for treating the D283 Med cells due to the predominant cytotoxic effect on the cell populations at higher concentrations [34]. In all experiments, the cells were seeded into Petri dishes or culture flasks 24 h prior to the treatment. Untreated cells were used as controls in all experiments.

### Expression profiling

The total RNA of the treated cell populations was isolated using the GenElute™ Mammalian Total RNA Miniprep Kit (Sigma), and its concentration and integrity were determined using a spectrophotometer. This isolation of RNA was performed at day 3 after treatment. The conversion of the experimental RNA into cDNA and its further transcription and biotin-UTP labeling was performed using TrueLabeling-AMP™ 2.0 cRNA (SABiosciences, Frederick, MD, USA). After purification with the SuperArray ArrayGrade cRNA Cleanup Kit, the labeled target cRNA was hybridized to Human Cancer OHS-802 Oligo GEArray membranes that profile 440 genes (both SABiosciences). The expression levels of each gene were detected via chemiluminescence using the alkaline phosphatase-conjugated streptavidin substrate. The membranes were then recorded using the MultiImage™ II Light Cabinet DE- 500 (Alpha Innotech, CA, USA). The image data were processed and analyzed using the GEArray Expression Analysis Suite software (SABiosciences) with background subtraction. All data were standardized as a ratio of the gene expression intensity to the mean expression intensity of the selected *HSP90AB1* housekeeping gene, which was chosen using the GeNorm [35] and NormFinder [36] software tools. Standardized spot intensity ratios (treated/control samples) were calculated and data filtering criteria were as follows: genes with ratio higher than 2 were rated as upregulated and genes with ratio lower than 0.5 were rated as downregulated. The expression of the specific gene was evaluated as changed if the same trend of change, i.e. upregulation or downregulation was detected at least in four experimental variants (of six in total) regardless the concentrations used for treatment. Cluster analyses were performed using the GEArray Expression Analysis Suite software according to the design of the experiments, i.e., separately for each cell line and inhibitor type. DAVID software tool [37] was used for primary detection of relevant pathways.

### RT-PCR

The changes in the expression of the two selected candidate genes were evaluated using RT-PCR. The RNA was isolated as described above. A total of 0.25 μg RNA was then reverse transcribed using M-MLV reverse transcriptase (Top-Bio, Prague, Czech Republic) according to the manufacturer’s protocol. RT-PCR was performed on 4 μl cDNA using Taq DNA polymerase 1.1 (Top-Bio) with human primers for the *CDKN1A* and *GDF15* candidate genes as well as the *HSP90AB1* housekeeping gene (Table 1) in 20 μl of the reaction volume. The PCR reaction was performed with denaturation at 94°C for 4 min, annealing at 60°C for 30 s, and elongation at 72°C for 1 min (35 cycles for all primers) (Table 1). A total of 10 μl of the PCR product was loaded on the 1% agarose gel and examined using electrophoresis. The optical density was stained and quantified using ImageJ software [38], and the data were normalized to *HSP90AB1* expression.

**Table 1:** Sequences of primers used for RT-PCR

**Gene**	**Primer sequence**	**Product**
***CDKN1A***	F: 5′ TTA GCA GCG GAA CAA GGA GT 3′	225 bp
	R: 5′ GCC GAG AGA AAA CAG TCC AG 3′	
***GDF15***	F: 5′ CTC CAG ATT CCG AGA GTT GC 3′	169 bp
	R: 5′ AGA GAT ACG CAG GTG CAG GT 3′	
***HSP90AB1***	F: 5′ CGC ATG AAG GAG ACA CAG AA 3′	169 bp
	R: 5′ TCC CAT CAA ATT CCT TGA GC 3′	

## Results

**Figure 1:**
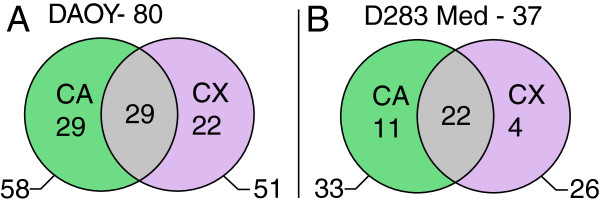
**Summary of detected changes in the expression of 440 cancer-related genes after the combined treatment of the Daoy (A) and D283 Med (B) cell lines.** The black circles indicate the total number of genes with changed expression after combinations of ATRA with CA or CX. The green areas indicate the number of genes with changed expression after combined treatment with ATRA and CA only. Similarly, the violet areas indicate the number of genes with changed expression after combined treatment with ATRA and CA only. The gray overlays indicate the number of genes that demonstrated changed expressions after treatment with ATRA in both combinations; i.e., with CA as well as with CX.

**Figure 2:**
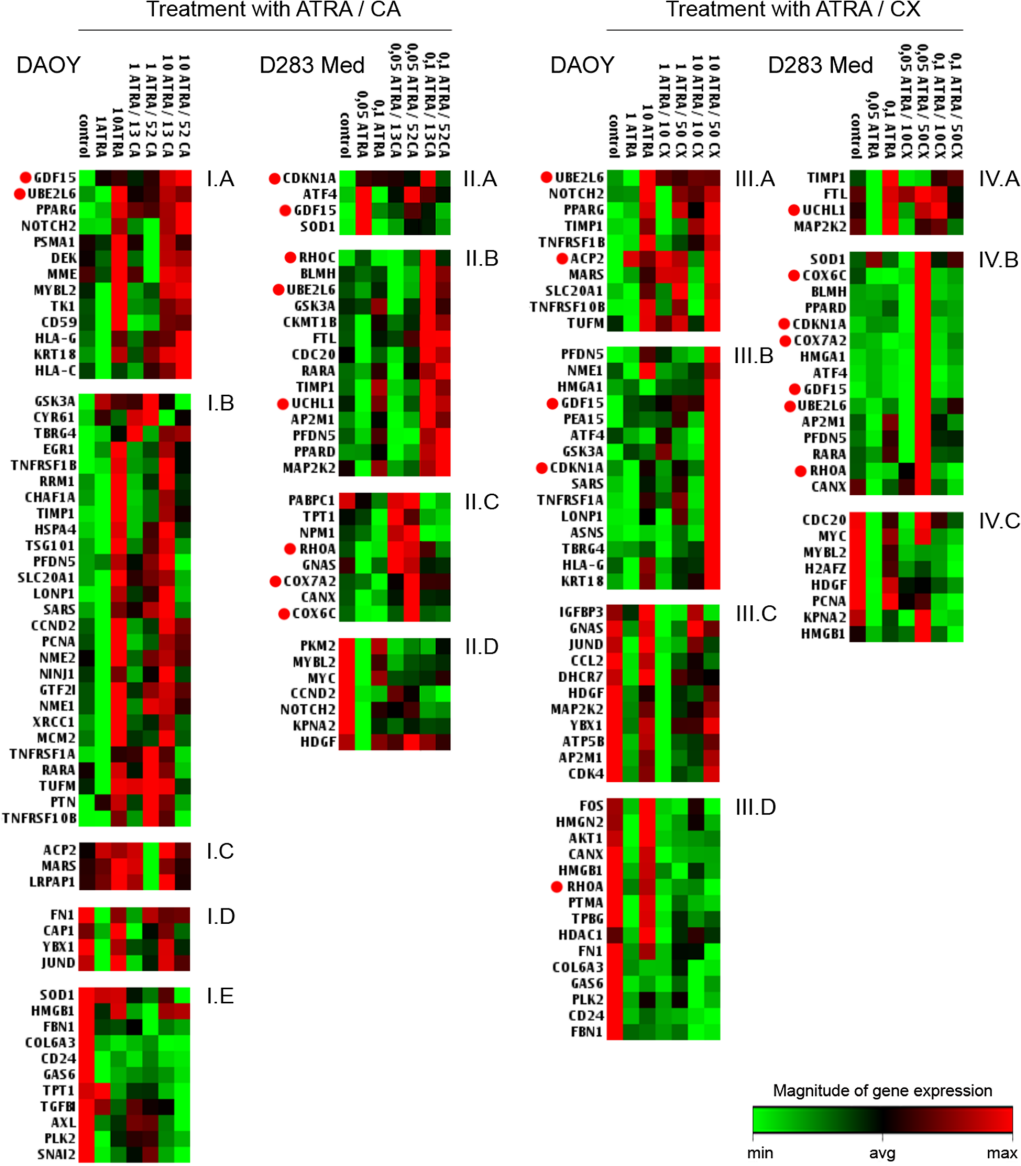
**Results of gene cluster analysis after the expression profiling of treated cells.** The genes were clustered according to the type of changes in the expression in the respective cell line (Daoy or D283 Med) after the combined treatment with ATRA and inhibitors (CA or CX). The cells were treated with ATRA alone or in combination with CA (an inhibitor of 5-LOX) or CX (an inhibitor of COX-2); numbers indicate the concentration in μM. The green color at the farthest left end of the color scale corresponds to the minimal value; the red color at the farthest right end of the color scale corresponds to the maximal value; the black color in the middle corresponds to the average value. Each of the other values corresponds to a certain color according to its magnitude. The colors are assigned according to the value of the particular gene expression in all samples in the respective experimental variant (I, II, III or IV). Genes discussed in the text in detail are highlighted by red dots.

The present study was focused on a detailed analysis of Daoy and D283 Med medulloblastoma cells after the combined application of ATRA and LOX/COX inhibitors. CA as the specific inhibitor of 5-LOX and CX as the specific inhibitor of COX-2 were used in these experiments. The changes in the expression of cancer-related genes were evaluated using expression profiling. Furthermore, a detailed analysis of the expression of five candidate genes was performed using RT-PCR to verify the microarray results. We used the same experimental design as our previous studies on neuroblastoma cells [20,29].In Daoy cells, changes in the expression of 80 cancer-related genes were detected after combined treatment with ATRA and inhibitors (Figure 1A). A total of 29 of these genes demonstrated changed expressions after combinations of ATRA and CA as well as of ATRA and CX. The expressions of another 29 genes were changed only after the combined treatment of ATRA and CA. A total of 22 different genes showed changes in expression after undergoing combined treatment with ATRA and CX (Figure 1A).In D283 Med cells, the expressions of 37 genes were changed (Figure 1B). Of these, 22 showed changes after combined treatment with ATRA and CA as well as with ATRA and CX. Changes in the expression of another 11 genes were identified after treatment with ATRA and CA only. Similarly, the expressions of 4 different genes were changed after treatment with ATRA and CX only (Figure 1B).The data achieved via expression profiling are presented after cluster analysis, which grouped the genes or gene groups by the type of changes in their expressions (Figure 2). Based on this analysis, three typical patterns of changes in gene expression are described:

(i.) Genes with strong concentration-dependent changes in their expressions. This pattern was typical in the I.A, II.A, III.A, and IV.A groups, in which the upregulation of gene expression was detected. This concentration-dependent pattern was also detected in the groups with downregulated gene expressions, i.e., in the I.E., II.D, III.D, and IV.C groups.

(ii.) Genes for which the expression was upregulated (groups I.B and II.C) or downregulated (group III.C) after treatment with lower concentrations of reagents. The use of higher concentrations had no or minimal influence on the expression of these genes.

(iii.) Genes with changes in their expressions after treatment with higher concentrations of reagents only. This pattern was characteristic in the II.B and III.B groups; however, such trends were also obvious in the I.A and IV.A groups.

Similarities in the gene expression changes are summarized in Table 2 and Table 3. The genes with changed expression in a particular cell line after combined treatment with ATRA and both inhibitors (CA or CX) are given: 8 genes were upregulated and 8 genes were downregulated in the Daoy cells; 16 genes were upregulated and 3 were downregulated in the D283 Med cells (Table 2). Two of these genes, *GDF15* and *UBE2L6*, were upregulated in both cell lines after all types of combined treatment (Table 2). Changes in gene expression after the same type of combined treatment (ATRA with CA or ATRA with CX) were identified in both cell lines; 2 of the genes were upregulated and 1 was downregulated after treatment with ATRA and CA, while 8 were upregulated and 1 was downregulated after treatment with ATRA and CX (Table 3).

**Table 2:** Genes with changed expression in a particular cell line (Daoy or D283 Med) after combined treatment with ATRA and both inhibitors (CA or CX)

**Daoy cell line**	
Upregulated:	*ACP2,* ***GDF15****, HLA-G, KRT18, MARS, NOTCH2, PPARG,* ***UBE2L6***
Downregulated:	*CD24, COL6A3, FBN1, GAS6, HMGB1, JUND, PLK2, YBX1*
**D283 Med cell line**	
Upregulated:	*AP2M1, ATF4, BLMH, CANX, CDKN1A, COX6C, COX7A2, FTL,* ***GDF15****, MAP2K2, PFDN5, PPARD, RARA, RHOA, SOD1, TIMP1,* ***UBE2L6****, UCHL1*
Downregulated:	*KPNA2, MYBL2, MYC*

The genes that were influenced via the combinations of both inhibitors are typed in bold.

**Table 3:** Genes with changed expression detected after the same type of combined treatment (ATRA with CA or ATRA with CX) in both cell lines (Daoy and D283 Med)

**ATRA/CA**	
Upregulated:	***GDF15, UBE2L6***
Downregulated:	*CCND2*
**ATRA/CX**	
Upregulated:	*ATF4, CDKN1A,* ***GDF15****, HMGA1, HMGB1, PFDN5, TIMP1,* ***UBE2L6***
Downregulated:	*AP2M1*

The genes that were influenced via the combinations of both inhibitors are typed in bold.

**Figure 3:**
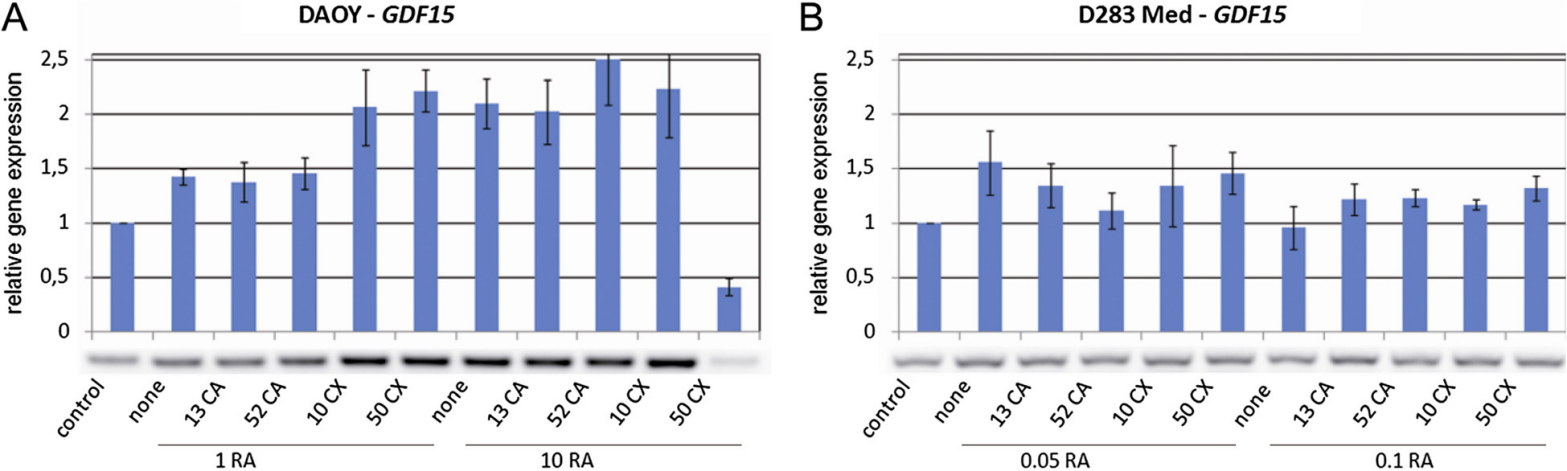
**Changes in the expression of the *GDF15 *gene in Daoy (A) and D283 Med (B) cells analyzed using RT-PCR.** The cells were treated with ATRA alone or in combination with CA (an inhibitor of 5-LOX) or CX (an inhibitor of COX-2); numbers indicate the concentrations of these compounds in μM. Respective numerical data from densitometry for three independent experiments are shown in the graphs: X-axis, treatment condition; Y-axis, relative gene expression. The data represent the means ± SD; they were normalized to the expression of the *HSP90AB1* housekeeping gene and were related to the untreated control cells. Representative electrophoresis is presented for each cell line.

**Figure 4:**
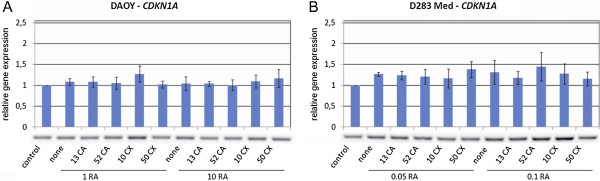
**Changes in the expression of the *CDKN1A *gene in Daoy (A) and D283 Med (B) cells analyzed using RT-PCR.** The cells were treated with ATRA alone or in combination with CA (an inhibitor of 5-LOX) or CX (an inhibitor of COX-2); numbers indicate the concentrations of these compounds in μM. Respective numerical data from densitometry for three independent experiments are shown in the graphs: X-axis, treatment condition; Y-axis, relative gene expression. The data represent the means ± SD; they were normalized to the expression of the *HSP90AB1* housekeeping gene and were related to the untreated control cells. Representative electrophoresis is presented for each cell line.

The analysis of the detected changes in gene expressions showed a concentration-dependent increase in the expression of genes known to be involved in the process of retinoid-induced differentiation and especially of the genes generally associated with the regulation of the cell cycle, of genes involved in mitochondrial metabolism, etc. The observed changes in the expressions of two selected candidate genes - *GDF15* (Figure 3) and *CDKN1A* (Figure 4) - were subsequently verified via RT-PCR.

## Discussion

At present, retinoids (powerful inducers of cell differentiation) have become a part of many therapeutic regimens in pediatric oncology, especially in the treatment of solid tumors of neurogenic origin [39-43]. In our previous experiments, we demonstrated the enhancement of the ATRA-induced differentiation of neuroblastoma cells via the combined application of LOX/COX inhibitors [20,29].

In our present study, we profiled the expression of 440 cancer-related genes in medulloblastoma cells after the same type of combined treatment (see above). Our results clearly confirmed our previous finding that ATRA-induced cell differentiation is enhanced via combined treatment with CA (inhibitor of 5-LOX) or with CX (inhibitor of COX-2) because the expression of the genes involved in the process of induced differentiation is increased in a concentration-dependent manner. A comparison of two established medulloblastoma cell lines showed a higher sensitivity in Daoy cells (compared with D283 Med cells) to the combined treatment with ATRA and inhibitors; nevertheless, our pilot experiments indicated that D283 Med cells were apparently more sensitive to the ATRA alone [34,44]. The same difference in sensitivity of Daoy and D283 Med cell lines to the ATRA was already described and the higher sensitivity of D283 Med cells is explained by expression of OTX2, a transcription factor, which was reported as a suppressor of neuronal differentiation in medulloblastoma cells [45].

The three patterns of changes in gene expression described here were very similar to our previous results on neuroblastoma cells [20]. In terms of the individual genes, we noted significant changes in the expression of the *GDF15*, *RHOA*, and *RHOC* genes. Although the expression of the *GDF15* gene was enhanced in both cell lines via combined treatment with ATRA and both inhibitors (Figures 2 and 3), changes in the expression of the *RHOA* and *RHOC* genes were detected in the D283 Med cells only (Figure 2). These two genes are members of the Rho GTPases family and are known to participate in cytoskeleton rearrangement and regulation processes such as changes in cell morphology during cell differentiation, proliferation and motility [46,47]. This finding is in accordance with our pilot study in which the decrease in proliferation activity and in the formation of multicellular aggregates was reported after the combined treatment (using CA or CX) of D283 Med cells [44].

The protein encoded by the *GDF15* gene is an important regulator of cell differentiation during embryonal development, especially in neural tissues [48]. This protein also has other important functions in the regulation of immune response or response to stress factors [49], and some studies have described its role as a paracrine mediator in the p53 cell signaling pathway that can inhibit cell proliferation and induce apoptosis [50,51]. Furthermore, the expression of *GDF15* correlated with the amount of the p21 protein that was encoded by the *CDKN1A* gene [50,51]. Although the overexpression of *GDF15* was detected in both cell lines after combined treatment with both inhibitors (Tables 2 and 3), we observed some differences between these cell lines. The expression of *GDF15* increased in Daoy cells in a concentration-dependent manner (Figures 2 and 3A), but the changes in *GDF15* expression in D283 Med cells were not so obvious (Figures 2 and 3B).

As mentioned above, some previous studies have demonstrated a correlation between the expressions of the *GDF15* and *CDKN1A* genes [50,51]. The *CDKN1A* gene encodes the p21 protein, which serves as an important regulator of the cell cycle progression via the inhibition of cyclin-dependent kinases. In our experiments, the expression of *CDKN1A* was enhanced in both cell lines after combined treatment with ATRA and CX, whereas the combination of ATRA and CA did not show a similar effect (Table 3, Figure 4). The increase in *CDKN1A* expression after treatment with retinoids was described in many human malignancies under *in vivo* and *in vitro* conditions: acute promyelocytic leukemia [52], acute T-lymphoblastic leukemia [53], pre-B lymphoma [54], hepatoblastoma [55,56], and neuroblastoma [57-60]. Our *in vitro* data confirmed these findings (especially in D283 Med cells) and also clearly showed that this effect of ATRA can be enhanced through its combined administration with CX (Figure 4B).

Furthermore, we detected the upregulated expression of the *UBE2L6* gene, which encodes a member of the E2 ubiquitin-conjugating enzyme family in both cell lines after combined treatment with both inhibitors (Tables 2 and 3). The overexpression of the *ACP2* gene encoding a beta-subunit of lysosomal acid phosphatase was observed in Daoy cells after combined treatment with both inhibitors (Table 2). The upregulated expression of both of these genes indicated an increased activity of proteasome in the treated cells. Higher proteasome activity was reported in breast carcinoma cells [61] as well as in acute promyelocytic leukemia cells [62,63] after treatment with retinoids. However, the relationship between retinoids and proteasome activity should be associated with resistance to retinoids [64]. This mechanism of resistance to retinoids, i.e., the increased activity of proteasome in Daoy cells, could be one of the possible explanations for the differing sensitivity demonstrated by these cell lines to ATRA treatment, as reported in our pilot study [44]. This hypothesis is also supported by the fact that the upregulation of the *UCHL1* gene encoding a deubiquitinating enzyme was detected in more sensitive D283 Med cells after combined treatment with both inhibitors (Table 2). Moreover, the protein encoded by this gene was found solely in mature neurons [65]; its increased expression in D283 Med, which is apparently enhanced via a combined treatment with ATRA and inhibitors, should indicate the neuronal differentiation of D283 Med after experimental treatment.

Higher sensitivity of D283 Med cells to the ATRA-induced neuronal differentiation is also indicated by the overexpression of two genes encoding subunits of cytochrome C oxidase, *COX6C* and *COX7A2*, after both types of combined treatment (Table 2). An increased mitochondrial activity after treatment with retinoids was reported in neuroblastoma cell lines [66], and it was demonstrated that more differentiated neuronal cells exhibit higher oxygen consumption rates as well as metabolic rates [67]. These findings thus support the presumed neuronal differentiation of D283 Med cells treated with ATRA and the capability of CA and CX to enhance this effect. Furthermore, a similar upregulation of these two genes was previously detected in neuroblastoma cell lines after the same type of experimental treatment [20].

The presented results are closely connected to our previously published studies reporting encouraging treatment responses to metronomic therapy in children suffering from some types of relapsed solid tumors with poor prognosis [42,43,68]. In these protocols, retinoids are administered in combination with celecoxib (as an anti-angiogenic agent) and several cytotoxic agents. The usefulness of this type of metronomic therapy was also demonstrated in patients with MBL [43,69,70]. In light of this, our experimental data clearly demonstrated that the combined administration of retinoids and celecoxib should also be beneficial in enhancing tumor cell differentiation. Furthermore, a very similar effect could be achieved through the dietary uptake of plant phenolic compounds including caffeic acid [71,72].

## Conclusion

To summarize, our results on two established medulloblastoma cell lines – Daoy and D283 Med – confirmed our previous findings in leukemia and neuroblastoma cells that the differentiating effects of ATRA should be enhanced in its combined administration with caffeic acid (an inhibitor of 5-LOX) or celecoxib (an inhibitor of COX-2). This effect was apparently achieved in both cell lines via the increased expression of genes encoding proteins participating in inducing the differentiation and cytoskeleton remodeling (GDF-15, Rho GTPases) or the p21 protein, which is an important regulator of the cell cycle and of proteins associated with proteasome activity. Furthermore, our results showed an important difference between the established MBL cell lines: the Daoy cells showed the same sensitivity as the cell lines that were derived from other types of pediatric solid tumors, but the D283 Med cells were significantly more sensitive to the treatment with ATRA alone (this effect was further enhanced via combined treatment with LOX/COX inhibitors). To clarify detailed mechanisms of such difference, additional experiments concerning more cell lines derived from various MBL subtypes are needed. Nevertheless, the obtained results confirmed our initial hypothesis regarding the common mechanism of enhancement in ATRA-induced cell differentiation in various types of pediatric solid tumors.

## Abbreviations

ATRA: All-trans retinoic acid; COX-2: Cyclooxygenase 2; DMEM: Dulbecco’s modified Eagle’s medium; DMSO: Dimethyl sulfoxide; 5-LOX: 5-lipoxygenase; MBL: Medulloblastoma.

## Competing interests

The authors declare that they have no competing interests.

## Authors’ contributions

PC carried out the experiments with the cell lines, performed the expression profiling and RT-PCR and drafted the manuscript. JN participated in manuscript preparation and the experiments concerning RT-PCR and expression profiling and also in manuscript preparation. MR participated in the experiments with the cell lines. KZ participated in the analysis of results and in manuscript preparation. JS coordinated this study and participated in manuscript preparation. RV conceived the study, participated in the analysis of results and drafted the manuscript. All authors read and approved the final manuscript.

## References

[B1] Gerber NU Mynarek M von Hoff K Friedrich C Resch A Rutkowski S Recent developments and current concepts in medulloblastoma Cancer Treat Rev 2014 40 356 365 10.1016/j.ctrv.2013.11.010 24389035

[B2] Massimino M Giangaspero F Garre ML Gandola L Poggi G Biassoni V Gatta G Rutkowski S Childhood medulloblastoma Crit Rev Oncol Hematol 2011 79 65 83 10.1016/j.critrevonc.2010.07.010 21129995

[B3] Dragnev KH Petty WJ Dmitrovsky E Retinoid targets in cancer therapy and chemoprevention Cancer Biol Ther 2003 2 S150 S156 14508093

[B4] Brtko J Dvorak Z Role of retinoids, rexinoids and thyroid hormone in the expression of cytochrome p450 enzymes Curr Drug Metab 2011 12 71 88 10.2174/138920011795016881 21401514

[B5] Dedieu S Lefebvre P Retinoids interfere with the AP1 signalling pathway in human breast cancer cells Cell Signal 2006 18 889 898 10.1016/j.cellsig.2005.08.001 16176868

[B6] Masia S Alvarez S de Lera AR Barettino D Rapid, nongenomic actions of retinoic acid on phosphatidylinositol-3-kinase signaling pathway mediated by the retinoic acid receptor Mol Endocrinol 2007 21 2391 2402 10.1210/me.2007-0062 17595318

[B7] Cruz FD Matushansky I Solid tumor differentiation therapy - is it possible? Oncotarget 2012 3 559 567 22643847 10.18632/oncotarget.512 PMC3388185

[B8] Hallahan AR Pritchard JI Chandraratna RA Ellenbogen RG Geyer JR Overland RP Strand AD Tapscott SJ Olson JM BMP-2 mediates retinoid-induced apoptosis in medulloblastoma cells through a paracrine effect Nat Med 2003 9 1033 1038 10.1038/nm904 12872164

[B9] Garattini E Gianni M Terao M Retinoids as differentiating agents in oncology: a network of interactions with intracellular pathways as the basis for rational therapeutic combinations Curr Pharm Des 2007 13 1375 1400 10.2174/138161207780618786 17506722

[B10] Andres D Keyser BM Petrali J Benton B Hubbard KS McNutt PM Ray R Morphological and functional differentiation in BE(2)-M17 human neuroblastoma cells by treatment with Trans-retinoic acid BMC Neurosci 2013 14 49 10.1186/1471-2202-14-49 23597229 PMC3639069

[B11] Maurer BJ Kang MH Villablanca JG Janeba J Groshen S Matthay KK Sondel PM Maris JM Jackson HA Goodarzian F Shimada H Czarnecki S Hasenauer B Reynolds CP Marachelian A Phase I trial of fenretinide delivered orally in a novel organized lipid complex in patients with relapsed/refractory neuroblastoma: a report from the New Approaches to Neuroblastoma Therapy (NANT) consortium Pediatr Blood Cancer 2013 60 1801 1808 10.1002/pbc.24643 23813912 PMC4066886

[B12] Patatanian E Thompson DF Retinoic acid syndrome: a review J Clin Pharm Ther 2008 33 331 338 10.1111/j.1365-2710.2008.00935.x 18613850

[B13] Kuendgen A Gattermann N Valproic acid for the treatment of myeloid malignancies Cancer 2007 110 943 954 10.1002/cncr.22891 17647267

[B14] Tomita A Kiyoi H Naoe T Mechanisms of action and resistance to all-trans retinoic acid (ATRA) and arsenic trioxide (As2O 3) in acute promyelocytic leukemia Int J Hematol 2013 97 717 725 10.1007/s12185-013-1354-4 23670176

[B15] Nowak D Stewart D Koeffler HP Differentiation therapy of leukemia: 3 decades of development Blood 2009 113 3655 3665 10.1182/blood-2009-01-198911 19221035 PMC2943835

[B16] Lo-Coco F Avvisati G Vignetti M Thiede C Orlando SM Iacobelli S Ferrara F Fazi P Cicconi L di Bona E Specchia G Sica S Divona M Levis A Fiedler W Cerqui E Breccia M Fioritoni G Salih HR Cazzola M Melillo L Carella AM Brandts CH Morra E von Lilienfeld-Toal M Hertenstein B Wattad M Lübbert M Hänel M Schmitz N Retinoic acid and arsenic trioxide for acute promyelocytic leukemia N Engl J Med 2013 369 111 121 10.1056/NEJMoa1300874 23841729

[B17] Haque A Banik NL Ray SK Emerging role of combination of all-trans retinoic acid and interferon-gamma as chemoimmunotherapy in the management of human glioblastoma Neurochem Res 2007 32 2203 2209 10.1007/s11064-007-9420-z 17676389

[B18] De los Santos M Zambrano A Aranda A Combined effects of retinoic acid and histone deacetylase inhibitors on human neuroblastoma SH-SY5Y cells Mol Cancer Ther 2007 6 1425 1432 10.1158/1535-7163.MCT-06-0623 17431121

[B19] Frumm SM Fan ZP Ross KN Duvall JR Gupta S VerPlank L Suh BC Holson E Wagner FF Smith WB Paranal RM Bassil CF Qi J Roti G Kung AL Bradner JE Tolliday N Stegmaier K Selective HDAC1/HDAC2 inhibitors induce neuroblastoma differentiation Chem Biol 2013 20 713 725 10.1016/j.chembiol.2013.03.020 23706636 PMC3919449

[B20] Chlapek P Redova M Zitterbart K Hermanova M Sterba J Veselska R Enhancement of ATRA-induced differentiation of neuroblastoma cells with LOX/COX inhibitors: an expression profiling study J Exp Clin Cancer Res 2010 29 45 10.1186/1756-9966-29-45 20459794 PMC2874523

[B21] Avis I Martinez A Tauler J Zudaire E Mayburd A Abu-Ghazaleh R Ondrey F Mulshine JL Inhibitors of the arachidonic acid pathway and peroxisome proliferator-activated receptor ligands have superadditive effects on lung cancer growth inhibition Cancer Res 2005 65 4181 4190 10.1158/0008-5472.CAN-04-3441 15899809

[B22] Kuo HC Kuo WH Lee YJ Wang CJ Tseng TH Enhancement of caffeic acid phenethyl ester on all-trans retinoic acid-induced differentiation in human leukemia HL-60 cells Toxicol Appl Pharmacol 2006 216 80 88 10.1016/j.taap.2006.04.007 16766008

[B23] Veselska R Zitterbart K Auer J Neradil J Differentiation of HL-60 myeloid leukemia cells induced by all-trans retinoic acid is enhanced in combination with caffeic acid Int J Mol Med 2004 14 305 310 15254783

[B24] Bell E Ponthan F Whitworth C Westermann F Thomas H Redfern CP Cell Survival Signalling through PPARdelta and Arachidonic Acid Metabolites in Neuroblastoma PLoS One 2013 8 e68859 10.1371/journal.pone.0068859 23874790 PMC3706415

[B25] Schroeder CP Kadara H Lotan D Woo JK Lee HY Hong WK Lotan R Involvement of mitochondrial and Akt signaling pathways in augmented apoptosis induced by a combination of low doses of celecoxib and N-(4-hydroxyphenyl) retinamide in premalignant human bronchial epithelial cells Cancer Res 2006 66 9762 9770 10.1158/0008-5472.CAN-05-4124 17018636

[B26] Simeone AM Li YJ Broemeling LD Johnson MM Tuna M Tari AM Cyclooxygenase-2 is essential for HER2/neu to suppress N- (4-hydroxyphenyl) retinamide apoptotic effects in breast cancer cells Cancer Res 2004 64 1224 1228 10.1158/0008-5472.CAN-03-2188 14973114

[B27] Liu JP Wei HB Zheng ZH Guo WP Fang JF Celecoxib increases retinoid sensitivity in human colon cancer cell lines Cell Mol Biol Lett 2010 15 440 450 10.2478/s11658-010-0016-2 20496179 PMC6275995

[B28] Soda M Hu D Endo S Takemura M Li J Wada R Ifuku S Zhao HT El-Kabbani O Ohta S Yamamura K Toyooka N Hara A Matsunaga T Design, synthesis and evaluation of caffeic acid phenethyl ester-based inhibitors targeting a selectivity pocket in the active site of human aldo-keto reductase 1B10 Eur J Med Chem 2012 48 321 329 22236472 10.1016/j.ejmech.2011.12.034

[B29] Redova M Chlapek P Loja T Zitterbart K Hermanova M Sterba J Veselska R Influence of LOX/COX inhibitors on cell differentiation induced by all-trans retinoic acid in neuroblastoma cell lines Int J Mol Med 2010 25 271 280 20043138

[B30] Nardini M Scaccini C Packer L Virgili F In vitro inhibition of the activity of phosphorylase kinase, protein kinase C and protein kinase A by caffeic acid and a procyanidin-rich pine bark (Pinus marittima) extract Biochim Biophys Acta 2000 1474 219 225 10.1016/S0304-4165(00)00009-X 10742602

[B31] Dandekar DS Lopez M Carey RI Lokeshwar BL Cyclooxygenase-2 inhibitor celecoxib augments chemotherapeutic drug-induced apoptosis by enhancing activation of caspase-3 and -9 in prostate cancer cells Int J Cancer 2005 115 484 492 10.1002/ijc.20878 15688368

[B32] Kang KB Zhu C Yong SK Gao Q Wong MC Enhanced sensitivity of celecoxib in human glioblastoma cells: Induction of DNA damage leading to p53-dependent G1 cell cycle arrest and autophagy Mol Cancer 2009 8 66 10.1186/1476-4598-8-66 19706164 PMC2741461

[B33] Graff J Skarke C Klinkhardt U Watzer B Harder S Seyberth H Geisslinger G Nusing RM Effects of selective COX-2 inhibition on prostanoids and platelet physiology in young healthy volunteers J Thromb Haemost 2007 5 2376 2385 10.1111/j.1538-7836.2007.02782.x 17916229

[B34] Gumireddy K Sutton LN Phillips PC Reddy CD All-*trans*-retinoic acid-induced apoptosis in human medulloblastoma: activation of caspase-3/poly(ADPribose) polymerase 1 pathway Clin Cancer Res 2003 9 4052 4059 14519626

[B35] Vandesompele J de Preter K Pattyn F Poppe B van Roy N de Paepe A Speleman F Accurate normalization of real-time quantitative RT-PCR data by geometric averaging of multiple internal control genes Genome Biol 2002 3 RESEARCH0034 12184808 10.1186/gb-2002-3-7-research0034 PMC126239

[B36] Andersen CL Jensen JL Orntoft TF Normalization of real-time quantitative reverse transcription-PCR data: a model-based variance estimation approach to identify genes suited for normalization, applied to bladder and colon cancer data sets Cancer Res 2004 64 5245 5250 10.1158/0008-5472.CAN-04-0496 15289330

[B37] Huang DW Sherman BT Lempicki RA Systematic and integrative analysis of large gene lists using DAVID bioinformatics resources Nat Protoc 2009 4 44 57 19131956 10.1038/nprot.2008.211

[B38] Schneider CA Rasband WS Eliceiri KW NIH Image to ImageJ: 25 years of image analysis Nat Methods 2012 9 671 675 10.1038/nmeth.2089 22930834 PMC5554542

[B39] Reynolds CP Lemons RS Retinoid therapy of childhood cancer Hematol Oncol Clin North Am 2001 15 867 910 10.1016/S0889-8588(05)70256-2 11765378

[B40] Reynolds CP Matthay KK Villablanca JG Maurer BJ Retinoid therapy of high-risk neuroblastoma Cancer Lett 2003 197 185 192 10.1016/S0304-3835(03)00108-3 12880980

[B41] Sterba J Contemporary therapeutic options for children with high risk neuroblastoma Neoplasma 2002 49 133 140 12097996

[B42] Sterba J Valik D Mudry P Kepak T Pavelka Z Bajciova V Zitterbart K Kadlecova V Mazanek P Combined biodifferentiating and antiangiogenic oral metronomic therapy is feasible and effective in relapsed solid tumors in children: single-center pilot study Onkologie 2006 29 308 313 10.1159/000093474 16874014

[B43] Zapletalova D Andre N Deak L Kyr M Bajciova V Mudry P Dubska L Demlova R Pavelka Z Zitterbart K Skotakova J Husek K Martincekova A Mazanek P Kepak T Doubek M Kutnikova L Valik D Sterba J Metronomic chemotherapy with the COMBAT regimen in advanced pediatric malignancies: a multicenter experience Oncology 2012 82 249 260 10.1159/000336483 22538363

[B44] Redova M Enhancement of differentiation inductors’ effect on the solid tumors model *in vitro* PhD thesis Brno 2010 Masaryk University, Department of Experimental Biology

[B45] Bai RY Staedtke V Lidov HG Eberhart CG Riggins GJ OTX2 represses myogenic and neuronal differentiation in medulloblastoma cells Cancer Res 2012 72 5988 6001 10.1158/0008-5472.CAN-12-0614 22986744 PMC4861240

[B46] David M Petit D Bertoglio J Cell cycle regulation of Rho signaling pathways Cell Cycle 2012 11 3003 3010 10.4161/cc.21088 22825247 PMC3442911

[B47] Bustelo XR Sauzeau V Berenjeno IM GTP-binding proteins of the Rho/Rac family: regulation, effectors and functions in vivo Bioessays 2007 29 356 370 10.1002/bies.20558 17373658 PMC1971132

[B48] Strelau J Strzelczyk A Rusu P Bendner G Wiese S Diella F Altick AL von Bartheld CS Klein R Sendtner M Unsicker K Progressive postnatal motoneuron loss in mice lacking GDF-15 J Neurosci 2009 29 13640 13648 10.1523/JNEUROSCI.1133-09.2009 19864576 PMC3320210

[B49] Mimeault M Batra SK Divergent molecular mechanisms underlying the pleiotropic functions of macrophage inhibitory cytokine-1 in cancer J Cell Physiol 2010 224 626 635 10.1002/jcp.22196 20578239 PMC2932466

[B50] Li PX Wong J Ayed A Ngo D Brade AM Arrowsmith C Austin RC Klamut HJ Placental transforming growth factor-beta is a downstream mediator of the growth arrest and apoptotic response of tumor cells to DNA damage and p53 overexpression J Biol Chem 2000 275 20127 20135 10.1074/jbc.M909580199 10777512

[B51] Yang H Filipovic Z Brown D Breit SN Vassilev LT Macrophage inhibitory cytokine-1: a novel biomarker for p53 pathway activation Mol Cancer Ther 2003 2 1023 1029 14578467

[B52] Ryningen A Stapnes C Paulsen K Lassalle P Gjertsen BT Bruserud O In vivo biological effects of ATRA in the treatment of AML Expert Opin Investig Drugs 2008 17 1623 1633 10.1517/13543784.17.11.1623 18922099

[B53] Luo P Lin M Chen Y Yang B He Q Function of retinoid acid receptor alpha and p21 in all-trans-retinoic acid-induced acute T-lymphoblastic leukemia apoptosis Leuk Lymphoma 2009 50 1183 1189 10.1080/10428190902934936 19557639

[B54] Bao GC Wang JG Jong A Increased p21 expression and complex formation with cyclin E/CDK2 in retinoid-induced pre-B lymphoma cell apoptosis FEBS Lett 2006 580 3687 3693 10.1016/j.febslet.2006.05.052 16765349

[B55] Lim JS Park SH Jang KL All-trans retinoic acid induces cellular senescence by up-regulating levels of p16 and p21 via promoter hypomethylation Biochem Biophys Res Commun 2011 412 500 505 10.1016/j.bbrc.2011.07.130 21843507

[B56] Park SH Lim JS Jang KL All-trans retinoic acid induces cellular senescence via upregulation of p16, p21, and p27 Cancer Lett 2011 310 232 239 10.1016/j.canlet.2011.07.009 21803488

[B57] Wainwright LJ Lasorella A Iavarone A Distinct mechanisms of cell cycle arrest control the decision between differentiation and senescence in human neuroblastoma cells Proc Natl Acad Sci U S A 2001 98 9396 9400 10.1073/pnas.161288698 11481496 PMC55432

[B58] Liu Y Encinas M Comella JX Aldea M Gallego C Basic helix-loop-helix proteins bind to TrkB and p21(Cip1) promoters linking differentiation and cell cycle arrest in neuroblastoma cells Mol Cell Biol 2004 24 2662 2672 10.1128/MCB.24.7.2662-2672.2004 15024057 PMC371129

[B59] Marzinke MA Clagett-Dame M The all-trans retinoic acid (atRA)-regulated gene Calmin (Clmn) regulates cell cycle exit and neurite outgrowth in murine neuroblastoma (Neuro2a) cells Exp Cell Res 2012 318 85 93 10.1016/j.yexcr.2011.10.002 22001116

[B60] Qiao J Paul P Lee S Qiao L Josifi E Tiao JR Chung DH PI3K/AKT and ERK regulate retinoic acid-induced neuroblastoma cellular differentiation Biochem Biophys Res Commun 2012 424 421 426 10.1016/j.bbrc.2012.06.125 22766505 PMC3668681

[B61] Son SH Yu E Ahn Y Choi EK Lee H Choi J Retinoic acid attenuates promyelocytic leukemia protein-induced cell death in breast cancer cells by activation of the ubiquitin-proteasome pathway Cancer Lett 2007 247 213 223 10.1016/j.canlet.2006.04.005 16740359

[B62] Isakson P Bjoras M Boe SO Simonsen A Autophagy contributes to therapy-induced degradation of the PML/RARA oncoprotein Blood 2010 116 2324 2331 10.1182/blood-2010-01-261040 20574048

[B63] Trocoli A Mathieu J Priault M Reiffers J Souquere S Pierron G Besancon F Djavaheri-Mergny M ATRA-induced upregulation of Beclin 1 prolongs the life span of differentiated acute promyelocytic leukemia cells Autophagy 2011 7 1108 1114 10.4161/auto.7.10.16623 21691148 PMC3242613

[B64] Ferry C Gaouar S Fischer B Boeglin M Paul N Samarut E Piskunov A Pankotai-Bodo G Brino L Rochette-Egly C Cullin 3 mediates SRC-3 ubiquitination and degradation to control the retinoic acid response Proc Natl Acad Sci U S A 2011 108 20603 20608 10.1073/pnas.1102572108 22147914 PMC3251120

[B65] Day IN Thompson RJ UCHL1 (PGP 9.5): neuronal biomarker and ubiquitin system protein Prog Neurobiol 2010 90 327 362 10.1016/j.pneurobio.2009.10.020 19879917

[B66] Schneider L Giordano S Zelickson BR SJ M AB G Ouyang X Fineberg N Darley-Usmar VM Zhang J Differentiation of SH-SY5Y cells to a neuronal phenotype changes cellular bioenergetics and the response to oxidative stress Free Radic Biol Med 2011 51 2007 2017 10.1016/j.freeradbiomed.2011.08.030 21945098 PMC3208787

[B67] Xun Z Lee DY Lim J Canaria CA Barnebey A Yanonne SM McMurray CT Retinoic acid-induced differentiation increases the rate of oxygen consumption and enhances the spare respiratory capacity of mitochondria in SH-SY5Y cells Mech Ageing Dev 2012 133 176 185 10.1016/j.mad.2012.01.008 22336883 PMC3357086

[B68] Malik PS Raina V Andre N Metronomics as maintenance treatment in oncology: time for chemo-switch Front Oncol 2014 epub ahead of print, doi: 10.3389/fonc.2014.00076 10.3389/fonc.2014.00076 PMC3989712 24782987

[B69] Choi LMR Rood B Kamani N La Fond D Packer RJ Santi MR MacDonald TJ Feasibility of metronomic maintenance chemotherapy following high-dose chemotherapy for malignant central nervous system tumors Pediatr Blood Cancer 2008 50 970 975 10.1002/pbc.21381 17941070

[B70] Sterba J Pavelka Z Andre N Ventruba J Skotakova J Bajciova V Bronisova D Dubska L Valik D Second complete remission of relapsed medulloblastoma induced by metronomic chemotherapy Pediatr Blood Cancer 2010 54 616 617 19967772 10.1002/pbc.22382

[B71] Jaganathan SK Mandal M Antiproliferative effects of honey and of its polyphenols: a review J Biomed Biotechnol 2009 2009 830616 19636435 10.1155/2009/830616 PMC2712839

[B72] Korkina LG Phenylpropanoids as naturally occurring antioxidants: from plant defense to human health Cell Mol Biol (Noisy-le-Grand) 2007 53 15 25 17519109

